# Genetic and behavioural factors affecting interpopulation colour pattern variation in two congeneric chameleon species

**DOI:** 10.1098/rsos.231554

**Published:** 2024-01-17

**Authors:** Tammy Keren-Rotem, Devon C. Main, Adi Barocas, David Donaire-Barroso, Michal Haddas-Sasson, Carles Vila, Tal Shaharabany, Lior Wolf, Krystal A. Tolley, Eli Geffen

**Affiliations:** ^1^ Ecology Department, Israel Nature and Parks Authority, Jerusalem, Israel; ^2^ Centre for Ecological Genomics and Wildlife Conservation, University of Johannesburg, Auckland Park Campus, Johannesburg, South Africa; ^3^ San Diego Zoo Wildlife Alliance, Escondido, CA, USA; ^4^ Wildlife Conservation Research Unit, University of Oxford, Oxford, UK; ^5^ Asociación Herpetológica Fretum Gaditanum, Jerez de la Frontera, Spain; ^6^ School of Zoology, Tel Aviv University, Tel Aviv, Israel; ^7^ The Faculty of Engineering, Tel Aviv University, Tel Aviv, Israel; ^8^ School of Computer Science, Tel Aviv University, Tel Aviv, Israel; ^9^ Doñana Biological Station (EBD-CSIC), Seville, Spain; ^10^ Kirstenbosch Research Centre, South African National Biodiversity Institute, Cape Town, South Africa

**Keywords:** crypsis, communication, social state, alternative mating strategy, Chamaeleonidae

## Abstract

We conducted a study on interpopulation variation of colour patterns in two congeneric chameleon species, which have an analogous life history. Both species are able to rapidly change colour pattern, and their context-dependent colour patterns often vary across a wide geographical range. Specifically, we tested four hypotheses that can explain the observed interpopulation variation of colour patterns by a series of behavioural field trials where the colour patterns of individuals were recorded and later analysed by a deep neural network algorithm. We used redundancy analysis to relate genetic, spectral and behavioural predictors to interpopulation colour pattern distance. Our results showed that both isolation by distance (IBD) and alternative mating tactics were significant predictors for interpopulation colour pattern variation in *Chamaeleo chamaeleon* males. By contrast, in *Chamaeleo dilepis*, the interpopulation colour pattern variation was largely explained by IBD, and evidence for alternative mating tactics was absent. In both chameleon species, the environmental colours showed no evidence of influencing chameleon interpopulation colour pattern variation, regardless of sex or behavioural context. This contrasting finding suggests that interpopulation context-dependent colour pattern variations in each species are maintained under a different set of selective pressures or circumstances.

## 1. Introduction

Geographical variation in animal body colours (i.e. colour polymorphism) is a well-documented phenomenon (reviewed by Cuthill [1]), which has been extensively studied in birds (e.g. ventral colour of swallows: [2,3]) and in lizards [4–6]. In lizards, studies have shown that different populations of the same species exhibit dramatic variations in dorsal body colour, associated with different geographical environments, substrate colours and thermal environments [7,8]. The lizard dorsal body coloration is generally presumed to be an adaptation for crypsis, reducing visibility to avian predators [4–6,9,10], whereas the colours of the ventral and lateral sides are associated with social signalling among conspecifics [6,11]. Because the dorsal coloration in lizards is subjected to intense selection from avian predation, it is tightly correlated with the colours of the environment they inhabit (e.g. [9,12]). These colour patterns probably evolved to fit those environments in which lizards are most exposed to visual-based predators; for example, during basking or when foraging on exposed surfaces. Thus, populations of the same species living in different habitats demonstrate different dorsal patterns, which correspond to the colours and patterns of their environment's surface. Owing to intense selection pressure on the dorsal side, dorsal colour patterns are less influenced by geographical or genetic distances among lizard subpopulations. By contrast, colour patterns on the lizard's lateral and ventral sides, which evolved under sexual selection, are not visible to avian predators but are visible to conspecifics, and thus are used for intraspecific social communication [13]. In this context, it is interesting that in many lizard species, despite the lateral and ventral coloration of males being selected for attention grabbing, females do not discriminate between mates on the basis of colour patterns ([14–16]; but see [17]).

In lizards, the changing of body colour by an individual reflects a trade-off between two major selection pressures: camouflage and social signalling [13]. Some lizard species, including chameleons, anole lizards and several agamid species, have resolved this trade-off by means of a rapid and temporary change in body colour for both purposes [13]. Chameleons, unlike other colour-changing lizard species, are characterized by lateral body compression. Thus, colour patterns are displayed on the lateral sides and colour patterns both for background matching and for social signalling are displayed on the same body plane [18,19]. Chameleons can alternate between several colour patterns during social encounters, much like an electronic billboard that alternates between advertisements, entirely replacing one colour pattern with another [18,19]. Furthermore, males of *Chamaeleo chamaeleon* have two social states (i.e. female-guarding and sneaker). Males of the female-guarding state display a distinct body colour appearance throughout the breeding season to prospective female mates and other competing males [18]. Thus, both male types advertise their mating strategy via their body colour pattern, as in several other lizard species [20,21]. Honesty of colour signals in chameleons is maintained by physical aggression (i.e. social cost; [22]). Finally, some chameleon species show clear preference for sexual signalling over crypsis during mating encounters, and in some species this preference is also driven by habitat [13,18].

Chameleons can shift among several different colour patterns by means of a novel organization of iridophores into two superposed dermal layers. This lattice of small guanine nanocrystals enables some species to present both complex camouflage patterns and spectacular social displays on the same body plane [23,24]. Some colour patterns are used for short-term social displays (i.e. rapid colour change; [25–27]), while others are distinctive long-term social status colour patterns, displayed indefinitely [18]. Further, in some chameleon species, the ability to shift between contextual colour patterns results in several distinctive key body patterns. This makes chameleons an ideal study system to assess the factors that affect colour polymorphism among different populations within a species.

In this study, we focussed on two chameleon species, *Chamaeleo chamaeleon* and *Chamaeleo dilepis*, both able to change from one colour pattern to another in a matter of seconds, and whose contextual colour patterns often vary across a wide geographical range [18,19,28]. We selected these species because their populations occupy diverse vegetation, habitat and soil types, and the geographical distances between populations vary considerably. To explain the presence of local colour pattern phenotypes within chameleon species, we posited four non-exclusive hypotheses.

### 1.1. The genetic isolation hypothesis

Geographical distance often correctly reflects the level of isolation between populations: the greater the geographical distance, the less likelihood of genetic exchange [29,30]. This general understanding implies that genetic exchange between distant populations will be negligible, and that such distant populations are more likely to be phenotypically different, owing to genetic drift, compared to more geographically proximate populations. Consequently, if intraspecific differences among chameleon populations are genetically based, we predict a correlation between geographical or genetic distances and the distance between homologous colour pattern phenotypes. Accordingly, under the same contextual conditions, neighbouring chameleon populations on similar substrates are predicted to display a similar colour pattern, whereas distant populations should display vastly different colour patterns. We used the genetic distance between haplotypes to access the genetic difference between the sites we sampled (genetic principal component 1 (PC1), PC2).

### 1.2. The background matching hypothesis

Chameleons, and several other lizard species, are known for their background matching ability, with individuals altering their colour pattern to match that of their immediate environment [9,19,31]. Chameleons often colour match to the substrate, such as open ground, tree bark or leaves in their vicinity, an action that increases concealment and reduces detection by predators. Populations dwelling in habitats where light colours dominate (i.e. light coloured soils and trees) are predicted to possess colour pattern phenotypes that are largely composed of light colours, whereas populations occupying darker environments (i.e. dark coloured soils and trees) will possess phenotypes dominated by dark colour patterns. According to this hypothesis, we predict a correlation between environmental colours and chameleon colour pattern phenotypes. We quantify background colour by measuring vegetation and ground colour at the sampling locations (ground colour PC1, vegetation colour PC1).

### 1.3. The habitat selection hypothesis

In addition to the matching of background colour for camouflage, differing habitat types may exert distinct selection pressures. Individuals choosing among habitats should occupy those habitats that maximize their fitness [32]. Both chameleon species in this study occur in diverse habitat types, from dry forests to savannah and desert environments [28]. Populations in different habitats are subject to variation in climatic regime, vegetation structure, predator composition and prey-type availability. Habitat-related variation may engender differences in life-history strategies and behaviour, which could be partly expressed in body colour variation. For example, chameleons occupying darker habitats (i.e. dense forests) are more likely to use brighter and more contrasting colours for communication [33]. We quantify habitat type by measuring elevation and soil composition at the sampling locations (habitat PC1, PC2).

### 1.4. Alternative mating tactics hypothesis

A completely different process that is related to sexual selection may also contribute to the polymorphism among populations. The evolution of alternative male phenotypes is driven by male–male competition for access to reproductive females [34,35]. In some lizards, the dominant morph is larger and more aggressive, and the subordinate morph employs sneaker tactics, and these two tactics are manifested as colour morphs [20,21,36]. Keren-Rotem *et al*. [18] found that males of *C. chamaeleon* employ two alternative mating tactics. Large males are bright green in colour and often mate-guard receptive females. By contrast, small males are brown and patchy, resembling the colour of females, and behave as sneakers. This correlation between an individual's body size (i.e. snout-vent length (SVL)) and its body colour pattern may suggest the presence of alternative communication tactics between individuals or male alternative mating tactics [18]. Because colour patterns associated with alternative mating strategies may appear arbitrary with respect to the environment, males in different colour patterns may evolve in different populations. We quantify body size by measuring SVL of each individual.

Our goal was to determine whether the interpopulation variation in colour polymorphism is associated with genetic drift, habitat selection, background matching or social signalling. In *C. chamaeleon*, short-term colour patterns (i.e. rapid colour change) are used for social communication (e.g. during agonistic interactions and mating events) and long-term colour patterns are used both for crypsis and for social communication [18]. This species also has a distinct mating season where the need for social communication via colour change is prioritized over colour matching [19]. Given previous findings on several chameleon species [9,10,19,37], here we predicted that colour polymorphism among populations associated with crypsis will correlate with the local environment (the habitat selection and background matching hypotheses). By contrast, polymorphism among populations in colour patterns associated with social signalling will tend to show low correlation with geographical distance or habitat similarity.

## 2. Methods

### 2.1. Study design and field protocol

**Figure 1.**
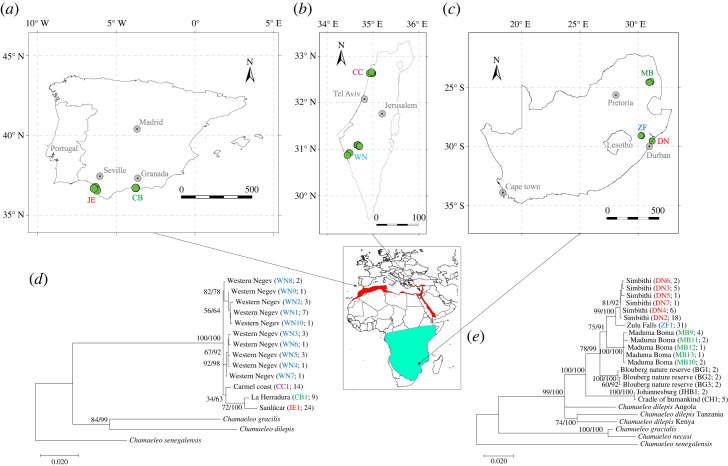
Distribution map for *Chamaeleo chamaeleon* (red) and *Chamaeleo dilepis* (green), and the sites where individuals were sampled in Spain (*a*), Israel (*b*), and South Africa (*c*). Mitochondrial DNA phylogeny for *Chamaeleo chamaeleon* (*d*), and *Chamaeleo dilepis* (*e*). Sites names are denoted by the same name and colour on maps and trees. Node labels denote the bootstrap support for the maximum-likelihood tree, and the support values for the Bayesian tree. Haplotype name and sample size are denoted in parenthesis. *Chamaeleo senegalensis* was set as the outgroup.

To examine colour polymorphism in chameleons, we studied two species, *C. chamaeleon* and *C. dilepis*, both with wide distributions across various habitats and of various degrees of geographical isolation among populations (figure 1). *Chamaeleo chamaeleon* is distributed in the Middle East, North Africa and some regions and islands of the Mediterranean and southern Europe [28], occurring in xeric and Mediterranean habitats on shrubs, bushes and trees [38]. *Chamaeleo dilepis* has a wide distribution, occurring in trees, bushes and some forest types within the savannah biome of sub-Saharan Africa ([28,38]; figure 1).

We sampled *C. chamaeleon* individuals on two sites in Spain (2017–2018), separated by about 230 km, and on two sites in Israel (2010–2011, 2016–2017), separated by about 180 km. In South Africa, we sampled *C. dilepis* individuals at three sites (2016, 2018), separated by 100–550 km (figure 1). For both species, our sampling sites represented a collection of diverse habitats (table 1).

**Table 1.** Sampling sites and habitat composition for *Chamaeleo chamaeleon* and *Chamaeleo dilepis*. (Mean air temperature (°C; minimum, maximum) during the sampling months (data from National Oceanic and Atmospheric Administration, National Centers for Environmental Information).)

site	latitude, longitude	habitats	temperature
*Chamaeleo chamaeleon* (September–October)			
Carmel coast (Israel)	32°38' N, 34°58' E	coastal oak forest, coastal sand dunes	24.6 (21.0, 28.2)
Northwestern Negev (Israel)	31°05' N, 34°40' E	desert sand dunes	24.1 (17.5, 30.7)
La Herradura (Spain)	36°44' N, 3°46' W	coastal oak-pine forest	21.5 (16.7, 26.3)
Sanlúcar area (Spain)	36°78' N, 6°35' W	coastal sand dunes	22.0 (18.6, 25.3)
*Chamaeleo dilepis* (January-February)			
Simbithi	29°30' S, 31°12' E	KwaZulu-Natal coastal grassland	25.1 (21.3, 29.0)
Zulu Falls	29°04' S, 31°17' E	KwaZulu-Natal highland thornveld	22.5 (16.0, 29.1)
Maduma Boma area	24°31' S, 31°04' E	granite lowveld savannah	22.5 (18.1, 26.9)

Fieldwork at each locality was carried out during the breeding season, which is the optimal time of year for body colour pattern documentation, as during this period individuals display accentuated colour patterns for social signalling. For South Africa, the optimal sampling time is during January–February, while in Israel and Spain it is during September–October. We manually collected individual chameleons from the vegetation using a spotlight during night when they sleep and their bodies stand out against the background [18,19]. To minimize stress, chameleons were kept for less than 12 h in two individual 35 × 20 cm plastic baskets, held together by metal clips, inside a fully ventilated room. This procedure prevented predation and overheating, while keeping air temperature and humidity conditions similar to their natural environment.

### 2.2. Experimental set-up

We subjected each individual to two field trials. First, we let each individual walk along a 2 m long horizontal stick placed 1 m above the ground in an open sunny space. A multicolour plate as a standard (GretagMacBeth ColorChecker chart; [39]; electronic supplementary material, figure S1a) and a white ruler running along the horizontal stick, both visible to the observers, enabled colour calibration and SVL measurements ([18,19]; electronic supplementary material, figure S1a,b). The colour patterns on both lateral sides were documented using a high-resolution digital video and images for 20 min (electronic supplementary material, figure S1b). Second, we introduced another individual by placing it on the same stick, 50 cm away. As before, all behaviours and colour pattern changes were recorded using 20 min long videos (electronic supplementary material, figure S1c). Our study was designed to maximize the number of trials involving male–male and female–male interactions. In order to eliminate pseudo-replications and carry-over effects, no individual was used more than once in social interaction trials. All animals were released back to their site of capture at the end of daily trials.

All trials were conducted in morning hours under natural light and ambient temperatures, which are typical ambient conditions for peak mating activity of these chameleon species (table 1), following our published protocol [18,19]. In our study design, we specifically selected populations that are roughly in the same climate zone (latitude range and range of temperature; table 1) within each species in order to reduce the effect of temperature regime. All the locations we sampled are in open habitats where direct sunshine is limitless.

### 2.3. Chameleon colour pattern and snout-vent length quantification

We employed computer vision techniques [18] to calculate similarity of colour patterns documented in digital photos between different populations and locations. To document the colour pattern of each animal in every trial, we used a high-resolution digital video camera (Panasonic HDC-TM300; 1920 × 1080 resolution for video and 11 megapixels for stills) placed on a tripod at a distance of 2 m from the focal animals. Photos were taken under natural sunlight, without a flash. Each photo included a colour standard in the form of a white ruler running along the horizontal stick and a standard colour board (GretagMacBeth ColorChecker chart; electronic supplementary material, figure S1a–c). We standardized the image white balance using the spectral reflectance of the colour board and Photoshop software (Adobe Systems, Inc.). Finally, we measured each individual's SVL from photos while walking along the horizontal stick ([18,19]; electronic supplementary material, figure S1b).

Next, we cropped every photo such that the image for analysis only contained the body pattern. Following the pre-processing steps, images were resized to a fixed size of 256 × 256 pixels and a signature for each image was obtained by applying a pre-trained Alexnet deep neural network [40]. Specifically, for each input image, the resulting activations of the penultimate layer of this neural network were recorded as the signature vector. This vector was used to represent the image in the next step of the process. We used the packages pytorch (version 1.7.1) and torchvision (version 0.8.2) for the Alexnet network calculations. The Euclidean distance between every two images was then computed, producing what we refer to as the ‘Alexnet distance matrix'. This matrix provided a measure of similarity between each pair of images and was used for further analysis.

In this study, we did not measure skin patterns reflected by UV light because, in our preliminary trials, we did not detect any reflective patterns under UV light (T. Keren-Rotem and E. Geffen 2010, personal observation). These preliminary observations are supported by an extensive comparative analysis among chameleon species showing that the species in the genus *Chamaeleo* lacks UV reflection [41].

### 2.4. Predictors of genetic divergence

A genetic distance is a representation of divergence time. To estimate the genetic distance, we took 3–4 drops of blood from captured individuals, by clipping the edge of a claw and gently pressing on the toe. Blood drops were stored for analysis on a Whatman FTA Classic Card. Blood stains on each paper were cut out and digested using an organic extracting protocol (based on the proteinase K—phenol DNA extraction procedure). For each sample, we sequenced two mitochondrial regions, *ND4* and *16S*, using the protocol in Main *et al*. [42]. These two mitochondrial DNA (mtDNA) regions have proved useful for delineating chameleon species [43]. We concatenated *ND4* (641 and 773 bp for *C. dilepis* and *C. chamaeleon*, respectively) and *16S* (497 and 490 bp for *C. dilepis* and *C. chamaeleon*, respectively) sequences into a single dataset and collapsed identical sequences into haplotypes using DnaSP (version 5.10; [44]). All haplotype sequences from each species were aligned using Clustal X [45] and checked for monophyly using the Bayesian and maximum-likelihood inference phylogenies (MrBayes 3.2, [46] and MEGA 7, [47]). Sequences from sister taxa, identical to Main *et al*. [42], were included to ensure both species formed monophyletic clades. The most appropriate substitution model for the phylogeny analyses was determined by the MODELS option in MEGA. We then used the aligned sequences to calculate a maximum-likelihood genetic distance matrix between all individuals of each species using the program MEGA. Finally, the maximum-likelihood genetic distance matrix was collapsed into one or two vectors using principal component analysis (PCA; table 2). Sequence accession numbers used in this study are MG952735.1-MG952756.1, OR230273-OR230365 and OR230438-OR230463.

**Table 2.** Predictors used in the distance-base redundancy analysis (dbRDA) for *Chamaeleo chamaeleon* (Cch) and *Chamaeleo dilepis* (Cdi). (The eigenvalues and cumulative percentage of the variation accounted for (in parenthesis) for the first and second principal components (PCs) are presented for each of the predictors. Owing to collinearity, geography PC1 was excluded from all models, and habitat PC2 was excluded from Cdi models.)

predictor	source	eigenvalue (cumulative %)
geography PC1	the first principal component of a table of geographical distances (km) between all individuals	Cch: 175.7 (99.8), Cdi: 101.1 (96.3)
genetics PC1, PC2	the first and second principal components of a table of maximum-likelihood genetic (mtDNA) distances between all haplotypes	Cch: 62.0, 4.3 (94.6), Cdi: 17.9 (94.0)
habitat PC1, PC2	the first and second principal components of elevation and the percentage of sand, silt and clay in the topsoil and subsoil at the individuals' capture locations	Cch: 4.1, 2.0 (87.3), Cdi: 4.2, 2.8 (99.9)
ground colour PC1	the first principal component of the RGB channels taken from the ground at the individuals' capture locations	Cch: 3.0 (98.6), Cdi: 2.8 (92.8)
vegetation colour PC1	the first principal component of the RGB channels taken from the vegetation at the individuals' capture locations	Cch: 2.9 (98.3), Cdi: 2.7 (89.4)
SVL	snout-vent length	

### 2.5. Predictors of habitat composition

We combined elevation above sea level and soil characteristics for classifying habitats at the sampling sites. We used the FAO/UNESCO Soil Map of the World (https://www.fao.org/soils-portal/data-hub/soil-maps-and-databases/faounesco-soil-map-of-the-world/en/) as a key for identifying soils, and for extracting the percentage of sand, silt and clay in the topsoil and subsoil at each capture location. PCA was used to reduce these seven variables into two vectors (i.e. Varimax rotation, eigenvalue ≥1) that together explained most of the habitat variation (table 2).

### 2.6. Predictors of vegetation and ground colour

For each study area, we obtained a Landsat 7 image [48] at a 30 m pixel resolution, containing the spatial locations of all found individuals. Satellite images for the three relevant study areas were obtained from the United States Geological Survey (USGS) website (www.usgs.gov) after undergoing initial processing and cross-calibration. We discarded images with cloud coverage, and selected images taken on average (±s.d.) 36.7 ± 14.8 days from the sampling date of each area.

We sampled the vegetation colour in a 300 × 300 m square around each chameleon location, and the ground colour at the most proximate 50 × 50 m square of bare ground for each location. For each such square, we used the mean pixel value of three colour bands (blue: band 2, green: band 3, red: band 4) from the relevant satellite image as the representation of dominant surrounding colours in that location. We used PCA to reduce the red/green/blue (RGB) channels into a single vector (i.e. varimax rotation, eigenvalue ≥1) of vegetation colour and a single vector of ground colour (table 2). We used quadratic discriminant function analysis (qDFA) to evaluate site variation in vegetation and ground colours.

The qDFA showed that within species, the ground and vegetation colours were clearly separated by the RGB channels (misclassification range 1.0–5.1%, Wilk's lambda (*Λ*) range 0.02–0.10, *p* ≤ 0.0001 in all cases; electronic supplementary material, figure S2a,b,d,e); thus the environmental colours at each site were different.

### 2.7. Statistical analysis

We used geography PC1 (i.e. the coordinates where each individual was found), genetics PC1 and PC2, habitat PC1 and PC2, ground colour PC1, vegetation colour PC1 and SVL as the predictors in the next analyses (eigenvalue ≥ 1, table 2). We tested the predictors for collinearity using correlations and the variation inflation factor (VIF). For both chameleon species' datasets, geography PC1 was highly correlated with genetics PC1 (*r* = 0.99 and *r* = 0.98). Geography PC1 was also highly correlated (*r* = −0.93) with habitat PC2 for the *C. dilepis* dataset. Therefore, we excluded geography PC1 from all the analyses and habitat PC2 from the analyses associated with the *C. dilepis* dataset. After excluding these two predictors, the VIF of all predictors in both datasets were ≤5.3 (acceptable level VIF ≤ 10).

We first evaluated the presence of site-specific chameleon patterns using quadratic qDFA and the Alexnet distance matrix. Specifically, we examined the *p*-value of Wilks’ lambda, 95% confidence ellipses, the percentage of misclassification of the model and the leave-one-out cross-validation (i.e. only one sample is used as a test set while the rest are used to train the model). The analysis was conducted separately for each sex and social context combination. The PCA and DFA analyses were conducted in JMP Pro (version 16, SAS Inc.).

Our field trials comprised six sex and social context combinations (i.e. single male on a pole, single female on a pole, male in a female–male match, female in a female–male match, male in a male–male match and female in a female–female match). We considered a single animal on a pole to represent a non-social scenario, where background-matching and habitat-related effects probably have a priority for the colour pattern used by chameleons. By contrast, the two-individual matches represent social scenarios, where the colour patterns used are probably more for social signalling and to reflect reproductive tactics, and less for camouflage.

To explain the individual pattern variation within each species, we used distance-based redundancy analysis (dbRDA; [49,50]) and the set of the above environmental and genetic predictors (table 2). All predictors were normalized prior to the analysis. The dependent variable in all the analyses was the Alexnet distance matrix between individuals, which measures the dissimilarity between individual colour patterns. The analysis was conducted separately for each sex and each social context combination. The dbRDA is an ordination procedure constrained to find linear combinations of the predictor variables that explain the greatest variation in the Alexnet distance matrix. We used marginal tests to determine the amount of variance explained by each predictor alone while ignoring all others; and sequential tests to determine the amount of variance explained by each individual predictor, added in a specified order. In the sequential tests, we added genetic components first, the habitat components second, colour components third, and the SVL last. *p*-values were evaluated using randomizations. The dbRDA models were calculated using PERMANOVA + (version 7, PRIMER-E Ltd.).

## 3. Results

Phylogenetic analysis of the individuals sampled at the study sites confirmed species monophyly. Animals sampled around the Mediterranean were all clustered as *C. chamaeleon* (i.e. 100% bootstrap support for the maximum-likelihood tree and 100% Bayesian support value), with a supported separation between populations from Israel and Spain (figure 1*d*). Animals sampled in South Africa were all clustered as *C. dilepis* (i.e. 99% bootstrap support for the maximum-likelihood tree and 100% Bayesian support value), with each locality clustered as a significantly different set of haplotypes (figure 1*e*). Overall, the phylogeny analysis provided strong evidence that, within chameleon species, the study locations constituted separate genetic populations.

**Figure 2.**
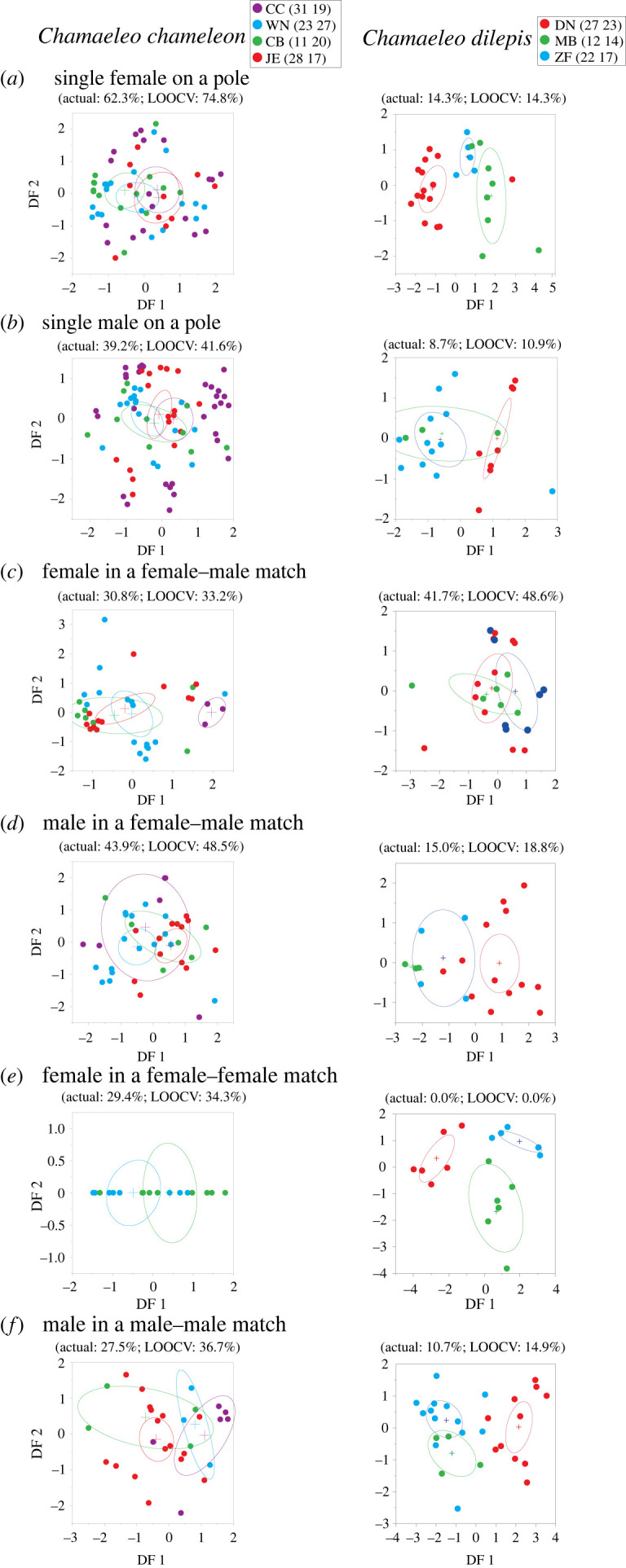
Discriminant function analysis (DFA) for the colour patterns of individuals (i.e. Alexnet distance) within *Chamaeleo chamaeleon and Chamaeleo dilepis*. The analysis was conducted for a single female on a pole (*a*), a single male on a pole (*b*), a female in a female–male match (*c*), a male in a female–male match (*d*), a female in a female–female match (*e*), and a male in a male–male match (*f*). Sites are denoted by letters and colour (Israel: CC, Carmel coast and WN, western Negev; Spain: CB, La Herradura and JE, Sanlúcar; South Africa: DN, Simbithi; MB, Maduma Boma and ZF, Zulu Falls), and the number of males and females sampled per site are in parenthesis following the site label. Actual and leave-one-out cross-validation misclassification rates are presented above each DFA plot. Site centroids (crosses) and 95% confidence interval ellipsoids are denoted by colour.

Regardless of sex and social contexts, the DFA for *C. chamaeleon* showed a poor correspondence between colour pattern and site (misclassification ranged 27–62%, Wilks lambda range 0.61–0.91, *F* ≤ 2.3, *p* ≥ 0.053; figure 2). By contrast, in *C. dilepis*, the DFA showed a clear separation by colour pattern between sites (misclassification range 0–15%, Wilks lambda range 0.07–0.58, *F* ≥ 3.0, *p* ≤ 0.03; figure 2), except for females in female–male matches (misclassification 41.7%, Wilks lambda range 0.83, *F* = 0.9, *p* = 0.46). The leave-one-out cross-validation misclassification values in all DFAs closely resembled the actual DFA misclassification values, indicating the signal strength in the data.

While *C. dilepis* females were significantly larger than males (*F*_1,108_ = 167.2, *p* < 0.0001) at all sites, individuals from Simbithi were overall significantly smaller (*F*_1,107_ = 48.4, *p* < 0.0001; electronic supplementary material, figure S2f) than other sites. By contrast, the *C. chamaeleon* females were significantly larger than males only at Sanlúcar (site * sex interaction; *F*_3,165_ = 5.8, *p* = 0.0009; electronic supplementary material, figure S2c).

**Figure 3.**
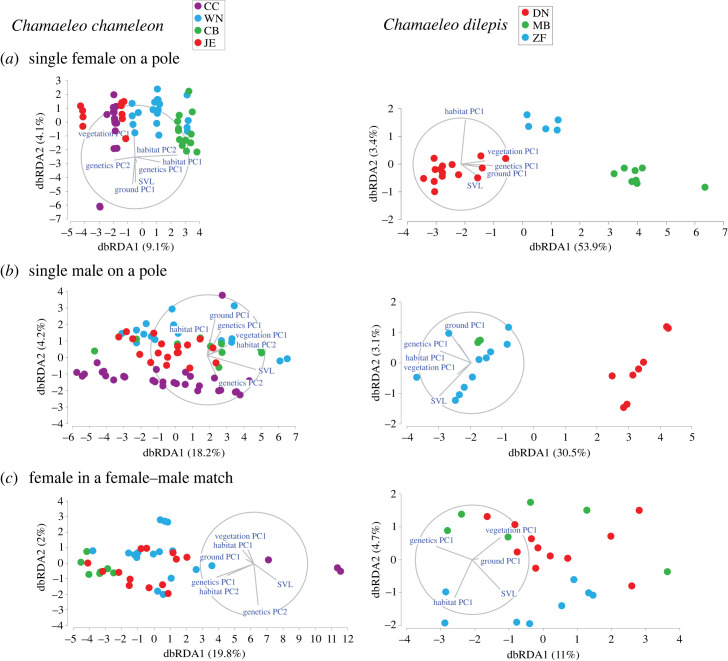

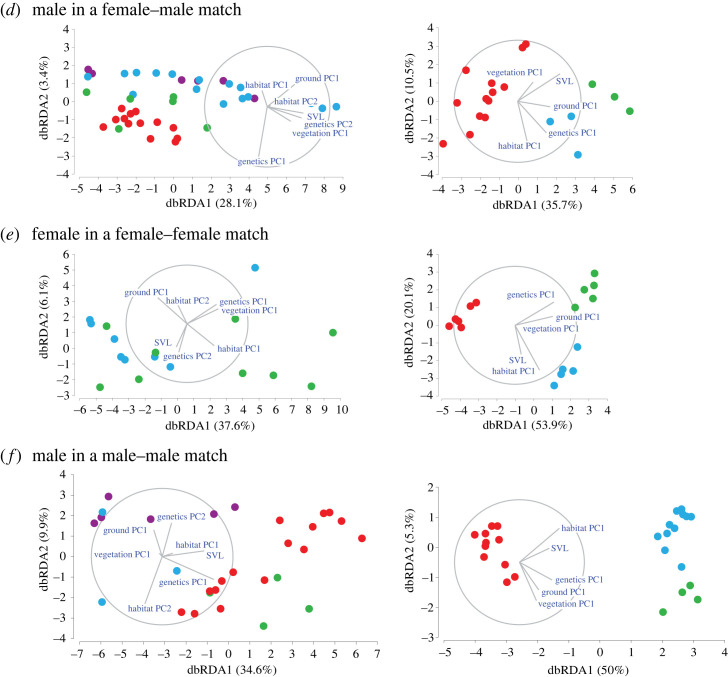
Distance-based redundancy analysis (dbRDA) plots for a single female on a pole (*a*), a single male on a pole (*b*), a female in a female–male match (*c*), a male in a female–male match (*d*), a female in a female–female match (*e*), and a male in a male–male match (*f*). Sites are denoted by letters and colour (Israel: CC, Carmel coast and WN, western Negev; Spain: CB, La Herradura and JE, Sanlúcar; South Africa: DN, Simbithi, MB, Maduma Boma and ZF, Zulu Falls). Rays indicate direction and magnitude of predictors. The amount of variation explained by each dbRDA dimension is indicated in parentheses.

Overall, the dbRDA models revealed that both genetic distance (Δ*R*^2^ range 0.16–0.22) and body size (Δ*R*^2^ range 0.10–0.15) were significant predictors for colour patterns of *C. chamaeleon* males (table 3 and figure 3). By contrast, we only observed weak evidence of an effect of ground or vegetation colour (*p*-values ranged 0.034–0.081) on the colour patterns of *C. chamaeleon* females. The colour patterns in *C. dilepis* were significantly explained by the genetic (Δ*R*^2^ range 0.17–0.46) and habitat predictors (Δ*R*^2^ range 0.07–0.35), while both environmental colours and body size showed no evidence of influencing chameleon colour patterns, regardless of sex or context (table 4 and figure 3). Finally, in *C. dilepis*, the significant effects of ground colour, vegetation colour, or body size, detected in the marginal tests, were all accounted for by the genetic and habitat predictors in the sequential tests (table 4).

**Table 3.** Distance-based redundancy analysis (dbRDA) effects of genetics PC1 and PC2, habitat PC1 and PC2, ground colour PC1, vegetation colour PC1, and snout-vent length (SVL) on colour pattern (AlexNet distance) of *Chamaeleo chamaeleon* individuals by social and sex categories. Net contribution (*ΔR*^2^), the cumulative variance explained (*Σ*R^2^) and *p*-value by permutations are presented for each model. Significant *p*-values are denoted in bold.

	marginal test				sequential model				
	*R*^2^	d.f.	*F*	*p*-value	*ΔR*^2^	*Σ*R^2^	d.f.	*F*	*p*-value
single male on a pole									
genetics PC1 and PC2	0.018	3,71	0.6	0.634	0.018	0.018	3,71	0.6	0.636
habitat PC1 and PC2	0.021	3,71	0.7	0.578	0.023	0.040	5,69	0.8	0.521
ground colour PC1	0.020	2,72	1.5	0.216	0.034	0.074	6,68	2.5	0.081
vegetation colour PC1	0.022	2,72	1.6	0.234	0.005	0.079	7,67	0.4	0.722
SVL	0.124	2,72	10.2	**0.001**	0.145	0.224	8,66	12.3	**0.001**
single female on a pole									
Genetics PC1 and PC2	0.020	3,58	0.6	0.669	0.020	0.020	3,58	0.6	0.667
habitat PC1 and PC2	0.081	3,58	2.6	**0.035**	0.071	0.091	5,56	2.2	0.079
ground colour PC1	0.001	2,59	0.0	0.968	0.004	0.095	6,55	0.3	0.779
vegetation colour PC1	0.006	2,59	0.3	0.736	0.028	0.123	7,54	1.7	0.197
SVL	0.010	2,59	0.6	0.567	0.008	0.131	8,53	0.5	0.571
male in a female–male match									
genetics PC1 and PC2	0.160	3,38	3.6	**0.011**	0.160	0.160	3,38	3.6	**0.013**
habitat PC1 and PC2	0.030	3,38	0.6	0.651	0.010	0.170	5,36	0.2	0.931
Ground colour PC1	0.091	2,39	3.9	**0.027**	0.029	0.199	6,35	1.3	0.283
vegetation colour PC1	0.089	2,39	3.8	**0.025**	0.006	0.204	7,34	0.2	0.776
SVL	0.082	2,39	3.5	**0.043**	0.110	0.314	8,33	5.3	**0.011**
female in a female–male match									
genetics PC1 and PC2	0.044	3,36	0.8	0.466	0.044	0.044	3,36	0.8	0.474
habitat PC1 and PC2	0.029	3,36	0.5	0.674	0.083	0.127	5,34	1.6	0.196
ground colour PC1	0.004	2,37	0.1	0.845	0.074	0.201	6,33	3.1	0.064
vegetation colour PC1	0.002	2,37	0.1	0.918	0.005	0.207	7,32	0.2	0.793
SVL	0.044	2,37	1.7	0.179	0.012	0.218	8,31	0.5	0.595
male in a male–male match									
genetics PC1 and PC2	0.221	3,26	3.7	**0.006**	0.221	0.221	3,26	3.7	**0.014**
habitat PC1 and PC2	0.099	3,26	1.4	0.235	0.088	0.310	5,24	1.5	0.187
ground colour PC1	0.068	2,27	2.0	0.137	0.021	0.330	6,23	0.7	0.489
vegetation colour PC1	0.034	2,27	0.9	0.410	0.016	0.346	7,22	0.5	0.575
SVL	0.139	2,27	4.3	**0.021**	0.098	0.445	8,21	3.7	**0.034**
female in a female–female match									
genetics PC1 and PC2	0.099	3,14	0.8	0.539	0.099	0.099	3,14	0.8	0.563
habitat PC1 and PC2	0.098	3,14	0.8	0.552	0.028	0.127	5,12	0.2	0.953
ground colour PC1	0.083	2,15	1.4	0.288	0.013	0.140	6,11	0.2	0.840
vegetation colour PC1	0.030	2,15	0.5	0.628	0.275	0.415	7,10	4.7	**0.034**
SVL	0.025	2,15	0.4	0.673	0.022	0.437	8,9	0.4	0.714

**Table 4.** Distance-based redundancy analysis (dbRDA) effects of genetics PC1, habitat PC1 and PC2, ground colour PC1, vegetation colour PC1, and snout-vent length (SVL) on colour pattern (AlexNet distance) of *Chamaeleo dilepis* individuals by social and sex categories. Net contribution (*ΔR*^2^), the cumulative variance explained (*ΣR*^2^) and *p*-value by permutations are presented for each model. Significant *p*-values are denoted in bold.

	marginal test				sequential model				
	*R*^2^	d.f.	*F*	*p­*-value	*ΔR*^2^	*ΣR*^2^	d.f.	*F*	*p*-value
single male on a pole									
genetics PC1	0.063	1,20	1.3	0.293	0.063	0.063	1,20	1.3	0.279
habitat PC1	0.136	1,20	3.2	0.063	0.225	0.288	1,19	6.0	**0.015**
ground colour PC1	0.121	1,20	2.8	0.092	0.009	0.297	1,18	0.2	0.779
vegetation colour PC1	0.072	1,20	1.6	0.216	0.005	0.302	1,17	0.1	0.898
SVL	0.254	1,20	6.8	**0.007**	0.034	0.336	1,16	0.8	0.449
single female on a pole									
genetics PC1	0.459	1,26	22.1	**0.001**	0.459	0.459	1,26	22.1	**0.001**
habitat PC1	0.033	1,26	0.9	0.409	0.071	0.530	1,25	3.7	**0.036**
ground colour PC1	0.418	1,26	18.7	**0.001**	0.007	0.537	1,24	0.4	0.684
vegetation colour PC1	0.351	1,26	14.1	**0.001**	0.028	0.565	1,23	1.5	0.216
SVL	0.228	1,26	7.7	**0.006**	0.008	0.573	1,22	0.4	0.651
male in a female–male match									
genetics PC1	0.239	1,17	5.4	**0.010**	0.239	0.239	1,17	5.4	**0.010**
habitat PC1	0.038	1,17	0.7	0.550	0.087	0.327	1,16	2.1	0.142
ground colour PC1	0.255	1,17	5.8	**0.003**	0.029	0.356	1,15	0.7	0.476
vegetation colour PC1	0.111	1,17	2.1	0.121	0.014	0.370	1,14	0.3	0.692
SVL	0.331	1,17	8.4	**0.002**	0.093	0.463	1,13	2.2	0.148
female in a female–male match									
genetics PC1	0.008	1,23	0.2	0.815	0.008	0.008	1,23	0.2	0.836
habitat PC1	0.034	1,23	0.8	0.447	0.029	0.038	1,22	0.7	0.513
ground colour PC1	0.002	1,23	0.0	0.972	0.046	0.084	1,21	1.0	0.351
vegetation colour PC1	0.024	1,23	0.6	0.586	0.048	0.131	1,20	1.1	0.319
SVL	0.027	1,23	0.6	0.547	0.025	0.156	1,19	0.6	0.591
male in a male–male match									
genetics PC1	0.173	1,24	5.0	**0.016**	0.173	0.173	1,24	5.0	**0.013**
habitat PC1	0.209	1,24	6.3	**0.008**	0.354	0.527	1,23	17.2	**0.001**
ground colour PC1	0.202	1,24	6.1	**0.006**	0.004	0.532	1,22	0.2	0.804
vegetation colour PC1	0.138	1,24	3.8	**0.026**	0.018	0.550	1,21	0.9	0.424
SVL	0.309	1,24	10.8	**0.002**	0.004	0.554	1,20	0.2	0.837
female in a female–female match									
genetics PC1	0.369	1,14	8.2	**0.004**	0.369	0.369	1,14	8.2	**0.003**
habitat PC1	0.199	1,14	3.5	**0.049**	0.339	0.708	1,13	15.1	**0.001**
ground colour PC1	0.460	1,14	11.9	**0.001**	0.002	0.710	1,12	0.1	0.923
vegetation colour PC1	0.147	1,14	2.4	0.104	0.002	0.711	1,11	0.1	0.937
SVL	0.138	1,14	2.2	0.116	0.029	0.741	1,10	1.1	0.354

## 4. Discussion

The ability to shift colour pattern and intensity varies between species and genera, but *C. chamaeleon* and *C. dilepis* are able to modify their body colour patterns greatly. Both species are of a similar size range, occupy relatively open habitats, and appear to possess an analogous life history [28,38]. Our analysis of intraspecific colour patterns by context revealed that, across the geographical range, *C. chamaeleon* colour pattern variations were independent of site. An opposite pattern was observed in *C. dilepis*, in which colour patterns were highly site-dependent. This contrasting finding suggests that intraspecific context-dependent colour pattern variations in each species are maintained under a different set of selective pressures or circumstances.

In lizards, the adaptive value of colour variation is associated with thermoregulation, camouflage, sexual selection and speciation [8,16,51,52]. Intraspecific colour polymorphism can also involve pleiotropy, epistatic interactions and stochastic processes [53,54]. Below we discuss the specific hypotheses for explaining the variation in colour patterns between chameleon populations.

Genetic differentiation often reflects isolation by distance (IBD) or physical barriers, and it is often the most parsimonious explanation for phenotypic variation between isolated populations [29]. Previous genetic studies suggest limited migration rates between chameleon populations. On the southern Iberian peninsula, the Mediterranean coast population of *C. chamaeleon* is closely related to North African populations along the Mediterranean coast, whereas the Atlantic coast population is closely related to populations along the Atlantic coast of North Africa [55]. Although these findings suggest a recent introduction, the two Iberian clusters remained separate for several hundred years, despite being geographically proximate. In Israel, *C. chamaeleon* populations form two genetic clusters on the northern and southern sides of the Jezreel Valley, which is about 12 km in width [56]. The divergence time between these clusters was estimated as three million years, which coincides with the flooding of the region from the Mediterranean Sea in the Pliocene. Surprisingly, this genetic division is still apparent regardless of the existence of chameleons and suitable habitat across the Jezreel Valley. Further evidence for limited migration rates comes from an extensive study of the panther chameleon (*Furcifer pardalis*) from Madagascar, which revealed strong genetic structure among geographically defined colour groups [57]. For this species, colour patterns were associated with genetic haplogroups that also occurred at different geographical locations. Our results provide strong evidence for an association between colour pattern and genetic distance, specifically among *C. dilepis* populations, suggesting that intraspecific differences in colour patterns are largely a result of IBD. *Chamaeleo dilepis* has an essentially continuous distribution in its South African range, with no physical or environmental barriers to immigration between sites. Evidence of IBD in *C. chamaeleon* was only partial, and differences in colour pattern were generally independent of geographical distance, despite the several thousand kilometres separating Iberian and Israeli populations.

In many lizard species, skin colour phenotypes are associated with environmental colours and habitat type for camouflage purposes [58,59]. We documented wide habitat variation between sites and showed that dominant ground and vegetation colours clustered by site in both South African and Mediterranean regions. For chameleons, which rely on camouflage for survival, we expected a strong effect of habitat and environment colour on individual colour patterns. However, we documented a significant habitat effect only in *C. dilepis* and found weak evidence of the effect of environmental colours on individual colour patterns, beyond the effect of habitat type, in both species. Thus, within the social context, the colour matching and habitat selection hypotheses are probably not the main drivers of interpopulation variation in colour patterns in either species.

Alternative mating tactics have been documented in a diverse range of vertebrates [34,60]. However, among chameleons, this has been documented only in *C. chamaeleon* [18]. Indeed, the lack of alternative mating tactics may be the expectation for chameleons in general but alternative mating tactics have not been studied in most chameleon species. Our analysis presents strong evidence for an association between body size and colour pattern in *C. chamaeleon* males, and the lack of such association in *C. dilepis* males. Interestingly, alternative mating tactics were only present in *C. chamaeleon* males, and not among *C. dilepis* males, despite these two species' similar life histories. Our results also support the idea that in a chameleon species with alternative mating tactics (i.e. *C. chamaeleon*), selection will favour multi-colour patterns for communication over environmental colour matching [19].

In conclusion, we showed that both IBD and alternative mating tactics were significant predictors for interpopulation colour pattern variation in *C. chamaeleon* males. By contrast, in *C. dilepis*, the interpopulation colour pattern variation was largely explained by IBD, and evidence for alternative mating tactics was absent. In both chameleon species, environmental colours showed no evidence of influencing chameleon interpopulation colour pattern variation, regardless of sex or behavioural context. This contrasting finding suggests that interpopulation context-dependent colour pattern variations in each species are maintained under a different set of selective pressures or circumstances. In order to better understand the reasons for the evolution of alternative mating tactics in *C. chamaeleon* and the possible lack of such behaviour in other chameleon species, future studies should also focus on modelling past demographic history and effective population size, and on ecophysiology.

## Data Availability

All the chameleon images used in this study are deposited in Dryad Digital Repository: https://doi.org/doi:10.5061/dryad.2547d7wwv [61]. Supplementary material is available online [62].
